# Enhancing the Oral Bioavailability of Candesartan Cilexetil Loaded Nanostructured Lipid Carriers: In Vitro Characterization and Absorption in Rats after Oral Administration

**DOI:** 10.3390/pharmaceutics12111047

**Published:** 2020-10-31

**Authors:** Walid Anwar, Hamdy M. Dawaba, Mohsen I. Afouna, Ahmed M. Samy, Mohammed H. Rashed, Abdelaziz E. Abdelaziz

**Affiliations:** 1 Department of Pharmaceutics, Faculty of Pharmacy, Al-Azhar University, Nasr City 11751, Cairo, Egypt or hamdy.dawaba@su.edu.eg (H.M.D.); mafouna@azhar.edu.eg (M.I.A.); amsahmed1951@yahoo.com (A.M.S.); 2 Department of Pharmaceutics, Faculty of Pharmacy, Sinai University, Al Qantarah Sharq 41636, Ismailia Governorate, Egypt; 3 Pharmacology and Toxicology Department, Faculty of Pharmacy, Al-Azhar University, Nasr City 11751, Cairo, Egypt; rashedmm1977@gmail.com; 4 Pharmaceutical Technology Department, Faculty of Pharmacy, Kafrelshiekh University, Kafrelshiekh 33516, Egypt; abdelazizabdrabo76@gmail.com

**Keywords:** Candesartan Cilexetil, nanostructured lipid carriers, oral bioavailability, lymphatic transport, cellular absorption

## Abstract

Candesartan Cilexetil (CC) is a prodrug widely used in the treatment of hypertension and heart failure, but it has some limitations, such as very poor aqueous solubility, high affinity to P-glycoprotein efflux mechanism, and hepatic first-pass metabolism. Therefore, it has very low oral bioavailability. In this study, glyceryl monostearate (GMS) and Capryol™ 90 were selected as solid and liquid lipids, respectively, to develop CC-NLC (nanostructured lipid carrier). CC was successfully encapsulated into NLP (CC-NLC) to enhance its oral bioavailability. CC-NLC was formulated using a hot homogenization-ultrasonication technique, and the physicochemical properties were characterized. The developed CC-NLC formulation was showed in nanometric size (121.6 ± 6.2 nm) with high encapsulation efficiency (96.23 ± 3.14%). Furthermore, it appeared almost spherical in morphology under a transmission electron microscope. The surgical experiment of the designed CC-NLC for absorption from the gastrointestinal tract revealed that CC-NLC absorption in the stomach was only 15.26% of that in the intestine. Otherwise, cellular uptake study exhibit that CC-NLCs should be internalized through the enterocytes after that transported through the systemic circulation. The pharmacokinetic results indicated that the oral bioavailability of CC was remarkably improved above 2-fold after encapsulation into nanostructured lipid carriers. These results ensured that nanostructured lipid carriers have a highly beneficial effect on improving the oral bioavailability of poorly water-soluble drugs, such as CC.

## 1. Introduction

Candesartan Cilexetil (CC) is inactive a prodrug ester of candesartan, readily and completely bioactivated via hydrolysis throughout absorption from the gastrointestinal (GI) tract to active form of candesartan [1,2].

CC is a selective AT1 subtype angiotensin II receptor antagonist and illustrates the width and the highest potency among the angiotensin II receptor antagonist used in the treatment of hypertension and heart failure. In clinical application, CC appears good safe profiles; wherein, there are no adverse effects on heart sings, kidney function, and other body organs [3,4].

The oral bioavailability of CC after oral administration showed a low value of around 15% in humans. The poor bioavailability of CC is related to some factors, such as a very poor aqueous (pka 6.0) solubility and high efflux mechanism through drug resistance pumps in epithelial cells of the gastrointestinal tract (P-glycoprotein). Moreover, CC undergoes hepatic first-pass metabolism [2,4,5,6,7,8].

Several studies with various oral dosage forms were achieved to overcome these problems and to improve the oral bioavailability of CC, including new solid dosage form [9], nanoparticles [10,11], niosomes [12], nanoemulsion [13], nanosuspension [14], SMEDDS [15], and solid dispersion [2].

Advanced development of a nanoparticulate system, lipid-based nanoparticulate systems LNS, such as solid–lipid nanoparticles SLNs and nanostructured lipid carrier (NLCs), can be developed. These delivery systems increase the absorption of lipophilic drugs into systemic circulation through enhancing its lymphatic transportation, and therefore, improve its oral bioavailability [16,17,18].

The lipid-based nanoparticulate systems LNS were stated as a replacement drug delivery system to conventional polymeric nanoparticles [19,20].

The advantages of lipid-based nanoparticulate systems over the polymeric nanoparticles depend on the unique lipid matrix composition, such as biocompatibility, biodegradability, protection of the encapsulated compound against enzymatic degradation [21], possibility incorporation both lipophilic and hydrophilic drugs, high entrapment efficiency, easy to scale-up and controlled release of drugs [18,19].

Besides, lipid-based nanoparticles are nanosized drug delivery system, which possesses the advantages of polymeric nanoparticles, liposomes, and emulsion.

Nanostructured lipid carriers are the second generation of lipid-based nanoparticles. NLCs were developed to solve the drawbacks of SLNs. Therefore, NLCs vary from SLNs by the feature of being smaller in size, better shelf-life stability, and better drug loading capacity [22].

NLCs are composed of a blend of solid lipids and liquid lipids in a suitable proportion. Presence of liquid lipids in solid matrix provide immobilization of drug and prevent the particle aggregation, due to solid matrix and therefore, inhibit expulsion of the drug [23,24]. Besides, the presence of liquid lipids increases the lipid matrix imperfections that lead to higher drug entrapment as compared to SLNs [18,25,26].

NLC can improve and enhance the oral bioavailability of loaded drugs by one or more of the following mechanisms:

(1) The mucoadhesion properties of NLCs to the intestinal wall;
(2) Emulsifying properties of surfactants used in formulation improve the permeability and solubility of the drug through the gastrointestinal tract membrane;
(3) The nanosize of NLCs contributes to the enterocyte’s surface to increase. Consequently, improve and enhance drug permeability across the intestinal membrane [27,28]. Meanwhile, the nanometric size of NLCs adhere to the gastrointestinal tract easily for a long period and to intervillar spaces lead to the mean residence time to increase associated with increased bioavailability of the loaded drug;
(4) Long carbon chain fatty acids of solid lipids used in NLCs formulation had an important role and more favor the lymphatic transport through chylomicron formation [16,17,29,30] leads to avoidance of first-pass metabolism [31].

The aim of this study was to design and development the NLCs delivery system as a potential oral formulation of CC to enhance its oral bioavailability by employing intestinal lymphatic transport.

Firstly, the selected glyceryl monostearate (GMS) and Capryol™ 90 as solid lipids and liquid lipids were used to develop CC-NLC formulation, which was prepared using a hot homogenization-ultrasonication technique. The Prepared CC-NLC was physically and chemically characterized.

The designed formulation was examined for pharmacokinetic behavior in rats to prove the validity and effectiveness of NLCs in improving the oral delivery of very poor aqueous-soluble CC, and the absorption of prepared CC-NLC in intestine and stomach were evaluated in comparison to a suspension of CC. Furthermore, the uptake of formulated CC-NLC in the Caco-2 cell monolayer was measured to confirm and identify the enhancement of penetration and absorption of CC-NLCs formulations.

## 2. Materials and Methods

### 2.1. Materials

The active CC was obtained as a gift from MEMPHIS, El-Amirya—Cairo—EGYPT. Cpryol™ 90 (propylene glycol monocaprylate type II) was kindly provided as a gift sample from Gattefosse (Saint Priest, France). Glyceryl monostearate (GMS), Lutrol^®^ F127, Carboxy methyl cellulose (CMC), and Nile red (log P = 3.10; octanol/water) were purchased from Sigma Aldrich, Inc. (St. Louis, MI, USA). Cremophor^®^ RH 40 (polyoxyl-40-hydrogenated castor oil) was supplied from BASF (Germany). Sodium hydroxide, potassium dihydrogen orthophosphate, Potassium Chloride, and Hydrochloric acid (HCL) were supplied by El-Nasr Pharm. Chem. Company, Cairo (Egypt). Membrane filter (0.22 µm) Millipore Iberica S.A.U.; Madrid (Spain). Methanol and Acetonitrile HPLC grade were purchased from Sigma Aldrich.

All the above materials were in analytical grade and were used without further purification.

### 2.2. Methods

#### 2.2.1. Preparation of NLC

##### Fabrication of Nanostructured Lipid Carrier (NLC)

Based on the previous study [32,33], a hot homogenization-ultrasonication technique was used for the preparation of CC nanostructured lipid carriers formulation (CC-NLC), but with some few modifications. Briefly, the mixture composed of liquid and solid lipids (5% *w*/*v*) was melted at 5 °C above the melting point of solid lipids. A fixed concentration of CC (5% *w*/*v* of lipids) was dissolved in the prepared oil phase (5% *w*/*v* mixture of solid and liquid lipids). The aqueous phase composed of Lutrol^®^ F127 and Cremophor^®^ RH 40 (2.5% *w*/*v*) was also heated at the same temperature and added dropwise to the lipid phase under magnetic stirring at 1500 rpm for 5 min. The resultant pre-emulsion was homogenized at 20,000 rpm for 10 min using an Ultra-Turrax T25 homogenizer (WiseMix™ HG15A, Daihan Scientific, Seoul, Korea). The resultant nanoemulsion (o/w) was subjected to probe sonication (ultrasonic processor, GE130, probe CV18) at a 60% amplitude for 10 min. The final NLC dispersion was left aside to reach room temperature.

##### Fabrication of Fluorescent Nile Red Loaded Nanostructured Lipid Carriers (NR-NLCs)

NR nanostructured lipid carriers (NR-NLC) were prepared by hot homogenization-ultrasonication technique as described above in the fabrication of nanostructured lipid carriers (NLC), but with substitution of the amount of CC by a certain amount of hydrophobic fluorescent dye Nile Red (NR) [34,35,36].

#### 2.2.2. Physicochemical Characterization of CC-NLC

##### Particle Size and Polydispersity Index

The mean diameter and polydispersity index of a particle of nanostructured lipid carriers loaded with CC was determined using a Zetasizer Nano-ZS (Malvern Instruments, Worcester, UK), equipped with a 10 mW He-Ne laser employing the wavelength of 633 nm and a back-scattering angle of 90 °C at 25 °C. Before photon correlation spectroscopic (PCS) analysis, CC-NLCs formulations should be diluted with a certain amount of double-distilled water (1:200) to get appropriate scattering intensity. The analysis, of particle size was determined using the Mie theory with the refractive index and absorbance of lecithin at 1.490 and 0.100, respectively [37].

##### Zeta Potential Analysis

The zeta potential of the developed NLC formulation was measured via electrophoretic mobility measurements using a Zetasizer Nano-ZS (Malvern Instruments, Worcester, UK). The zeta potential was calculated by applying the Helmholtz–Smoluchowski equation (*n* = 3) [38,39].

##### Encapsulation Efficiency (EE)

The encapsulation efficiency of CC into NLC formulation was measured by the indirect method by measuring the concentration of the free CC. Initially, 2 mL of prepared NLC formulation were centrifuged at 100,000 rpm for 1 h at 4 °C to evaluate the unentrapped CC using cooling ultracentrifuge (Beckman Instruments TLX-120 Optima Ultracentrifuge, USA) [40]. The aqueous layer was aspirated and filtered using Millipore^®^ membrane (0.2 μm) and diluted with an appropriate amount of methanol and measured by a UV-Vis spectrophotometer (Shimadzu, the model UV–1800 PC, Kyoto, Japan) at 254 nm to measure the free amount of CC. Consequently, the encapsulation efficiency of CC into NLC was determined through the following equation.

(1) $$EE\%=\;\frac{W_{i}-W_{f}}{W_{i}}\times 100$$

where, *Wi* = weight of the initial drug and *Wf* = weight of the free drug.

##### Determination of the Degree of Crystallinity and Polymorphism

The determination of the thermal behavior of the bulk excipients and identify the crystallinity and polymorphism of NLC formulations were performed using differential scanning calorimetry (DSC) technique. DSC thermograms of the following samples were observed using a differential scanning calorimeter (Perkin Elmer DSC Q2000 Exton, PA, USA).

The selected samples, GMS solid lipids, CC loaded NLC, blank of NLC, and CC powder in sufficient amount at least 3 mg were put in closed aluminum pans and heated with temperature ranged from 25 to 250 °C at a heating rate of 5 °C/min. The observed enthalpies, melting points, and the temperature of the transition state were recorded [21].

##### Fourier Transform Infrared Spectroscopy (FT-IR)

For identification presence of any interaction between CC and the other ingredients of CC-NLC, (FT-IR) investigation was performed for the residues obtained from CC loaded NLC, blank-NLC, pure powder of CC, pure powder of GMS in addition to physical mixture (CC and GMS).

Samples used for FT-IR examination were separately scanned over a wave number varied from 4000 to 650 cm^−1^ at a resolution of 4 cm^−1^ in the transmission mode using (FT-IR spectrometer Perkin Elmer, USA) [41].

##### Particle Morphology

The morphological structure of CC-NLC particles was observed visually using a transmission electron microscope (JOEL Transmission electron microscope (JTEM) model 1010 Tokyo, Japan) to investigate the outer surface of nanoparticles.

Furthermore, to clarify the presence of any colloidal species other than the expected NLCs. Initially, samples of CC-NLCs were diluted with (1:200) distilled water and carried on film-coated copper grids, and the samples were left to drying at room temperature overnight. Then, the prepared samples were examined under TEM [42,43,44].

#### 2.2.3. Stability Study of CC-NLC

The stability study of CC-NLC formulation was performed for three months. The selected formula CC-NLC was put in well-sealed amber glass bottles and stored at room temperature approximately (25 °C) and refrigerated temperature (2–6 °C). The stored samples were visually investigated for the presence of any physical changes as aggregation or separation. Furthermore, its examined periodically after one month, two months, and three months for particle size, polydispersity index (PDI), zeta potential (ZP), and encapsulation efficiency (EE) [45].

#### 2.2.4. In Vitro Drug Release Study

The dialysis bag diffusion technique was employed to assess the in vitro release of CC from CC suspension and CC-NLC. The receptor compartments contained 900 mL phosphate buffer solution (PBS; pH 6.8), and Tween 20 (0.7% *w*/*w*) was added to fulfill the sink conditions [46].

Cellulose membrane dialysis bags (MWCO-12,000, Sigma, Santa Clara, MO, USA) soaked in the dissolution media overnight before the experiment used to cover the donor compartment. Freshly prepared CC-NLC and CC suspension (equivalent to 2.5 mg of CC) diluted with 5 mL of dissolution media and which tightly closed from two sides by a thermo-resistant thread.

These bags were immersed in the Pharmatest^®^ Dissolution apparatus (six-spindle dissolution tester, type PTWII, Germany) preadjusted at 37 ± 2 °C and 100 rpm. At predetermined time intervals (0.5,1,2,4,6,8,10,12 and 24 h), two-milliliter sample was aspirated and replaced by an equal volume of media to maintain sink condition. UV-Vis spectrophotometer adjusted at 254 nm was to determine the amount released of CC from both NLC and suspension.

#### 2.2.5. In Vivo Studies

##### Absorption of CC Loaded NLC in the Gastrointestinal (GI) Tract

An in situ method was used for determining the absorption of CC-NLC in the gastrointestinal tract in rats, which described as following: the rats were prevented from eating overnight. After that, the rats were anesthetized by intraperitoneal injection of pentobarbital sodium (40 mg/kg).

A small opening was done in the abdomen, CC-NLC was injected directly in the stomach of rats by dose (10 mg/kg) after the ligation of the pylorus of the stomach to determine its absorption in the stomach. Similar to evaluate the absorption of CC-NLC in the intestine, CC-NLC was intraduodenal injected into rats at the same dose. The incision was stitched after the administration of CC-NLC.

Then, the body temperature of rats should be maintained by keeping it under infrared lamps. A volume of 1 mL of blood samples was collected in a heparinized tube at 0.25, 0.5, 0.75, 1, and 2 h, and plasma was separated by centrifugation at 10,000 rpm for 10 min [8,47].

Collected samples of plasma were stored at −20 °C until further investigations. The precipitation of plasma proteins of each sample was performed by adding 0.5 mL of acetonitrile, vortexed, and the supernatants were aspirated after centrifugation at 15,000 rpm for 10 min. Then, supernatants were filtered using polytetrafluoroethylene (PTFE) syringe filter (pore size 0.22 mm). Finally, 20 µL of the supernatant was analyzed for CC concentration using HPLC (Agilent 1100 liquid chromatography system, Agilent, Palo Alto, CA, USA).

A plasma sample (50 μL) was transferred to individual Eppendorf tubes, and 0.5 mL of acetonitrile was added and vortexed for 3 min for plasma protein precipitation. The supernatants were separated by centrifugation at 5000× *g* rpm for 15 min. Supernatants were filtered using polytetrafluoroethylene (PTFE) syringe filter (pore size 0.22 mm). Finally, 20 µL of the supernatant was analyzed for CC concentration using HPLC.

Chromatographic analysis was carried out on a liquid chromatography system. The chromatographic column used was Equisil BDS C18 (4.6 mm × 250 mm, 5 µ). The column temperature was kept at 25 °C, and the mobile phase consisted of acetonitrile-water (0.1% phosphoric acid and 0.1% triethylamine, pH 2.7) (35:65, *v*/*v*) [8]. The mobile phase was pumped with a flowrate of 1.0 mL/min. The eluent was detected using a UV detector at λ_max_ 254 nm.

##### Uptake of NLCs in Caco-2 Cell Monolayer

The uptake of Nile red loaded nanostructured lipid carrier (NR-blank-NLC) by Caco-2 cell monolayer was performed to clarify and confirm its absorption and penetration into the enterocytes. The human epithelial colorectal adenocarcinoma Caco-2 cells were used to study the cellular uptake efficiency of NLCs.

Caco-2 cells were maintained in a specific cultured flask 175 cm2 containing 20 mL of growth media of Roswell Park Memorial Institute media (RPMI), 100 u/mL of penicillin, 20 percent fetal bovine serum (FBS), and 50 μg/mL streptomycin. Then, the cells were cultured on sterile 22 mm2 cover slips (Harvard Apparatus, MA, USA) in six sterile well plates, at a density of 2.5 × 105 cells/mL and incubated at 37 °C for 24 h.

After an incubation period, the cells were treated with Nile Red-loaded blank NLCs for three hours at 2 mg/mL concentration in culture medium. At the end of the exposure, cells attached to cover slips were washed thrice with PBS, and the cover slips were then mounted on a glass slide with an anti-fade mounting medium containing 4’,6’-Diamidino-2-Phenylindole, dihydrochloride (DAPI) (Sigma-Aldrich, St. Louis, MO, USA), which was used as a counter stain of the nucleus and viewed with an epifluorescence microscope, Leica, DM 5500 B (Leica, Buffalo Grove, IL, USA) [8,48,49]. Data were captured digitally and quantified using the microscope provided software.

##### Pharmacokinetic Behavior in Rats

The pharmacokinetic study of CC-NLC and CC suspension were examined in rats (Sprague-Dawley rats) weighing 200 ± 20 g after oral administration. The rats were prevented from eating overnight and divided into two groups (*n* = 4). The first group received through oral route the free-CC suspension (CC dispersed in 0.5% sodium carboxymethyl cellulose solution, 0.5 mg/mL) by dose (10 mg per kg), and the second group was administered CC-NLC9 (2.5 mg/mL) at the same dose.

Blood samples (1 mL) were collected into tubes containing anticoagulant solution at chosen time intervals. Then the plasma was centrifuged at 10,000× *g* rpm for 10 min [8,47,50].

Collected samples of plasma were stored at −20 °C until further investigations. The precipitation of plasma proteins of each sample was performed by adding 0.5 mL of acetonitrile, vortexed, and the supernatants were centrifuged at 15,000× *g* rpm for 10 min. Supernatants were filtered using polytetrafluoroethylene (PTFE) syringe filter (pore size 0.22 mm). Finally, 20 µL of the supernatant was analyzed for CC concentration using HPLC.

The experimental protocol and animal used were approved by The Ethical Committee of Pharmacy college, Kaferelsheikh University (1 January 2017). The performed techniques were in agreement with the ARRIVE guidelines, European Union Directive 2010/63/EU, and the UK Animals (Scientific Procedures) Act, 1986 (ASPA).

#### 2.2.6. Statistics

Data obtained were evaluated as the mean ± SD, and the significance of difference was analyzed statistically by one-way of variance (ANOVA) using Graph-Pad Prisme software version 8 (Graph Pad, San Diego, CA, USA). *p* values ≤ 0.05 were reflected statistically significant.

## 3. Results and Discussion

### 3.1. Fabrication of Candesartan Cilexetil Nanostructured Lipid Carrier (CC-NLC)

GMS as solid lipids and Capryol™ 90 as liquid lipids and combination of Lutrol^®^ F127 with Cremophor^®^ RH as surfactants were chosen for preparation of CC-NLC. Lipid phase to surfactant concentrations were constant 5% (*w*/*w*) and 2.5% (*w*/*w*), respectively. Moreover, the CC concentration was fixed to 5% (*w*/*w*) of the lipid phase.

The lipid phase should not exceed 5% *w*/*w*. This remark is in agreement with studies conducted by Das et al. 2012 and Elbahwy et al. 2017 [51,52], who observed that an increasing the lipid concentration will lead to an enormous increase in the particle size.

Since the formulation was designed to be orally used, surfactants concentration has been established at 2.5% (*w*/*w*) [53].

CC-NLCs formulations were developed using homogenization, followed by probe sonication technique, and the developed formulations were subjected to physicochemical characterization.

### 3.2. Physicochemical Characterization of CC-NLC Formulation

#### 3.2.1. Particle Size, PDI, Zeta Potential, and Encapsulation Efficiency Measurements

The obtained findings revealed that the designed CC-NLC formulation was showed in the nanometer range (121.6 ± 6.2 nm). Further, the prepared CC-NLC possess acceptable values of particle size distribution and zeta potential (0.26 ± 0.03) and (−26.5 ± 2.9 mV), respectively, with high encapsulation efficiency (96.23 ± 3.14%) as depicted in Table 1.

The nanoparticles of a small size of the prepared formulation may be attributed to the presence of the combination of two surfactants, because of the combination of two or more surface-active agent exhibit to make blended surfactant films at the particles which wrapped the surface of particles efficiently to fabricate nanoparticles with small size [32,54,55].

Further, the poly dispersity index of formulated CC-NLC dispersion was in the optimum value, due to the formulation method used indicating fabricated nanoparticles in homogenous form and narrow size distributed [56].

The obtained Zeta potential value can be attributed to the structure of solid and liquid lipids [57] where GMS composed of triacylglycerols (5–15%), diacylglycerols (30–45%), and monoacylglycerols (40–55%). Therefore, it possesses a high content of partial emulsifying Glycerides (mono and Diglycerides) and the presence of non-esterified hydroxyl group of the glycerol, which gives certain polarity to this molecule that contributes to zeta potential [32,58]. On the other side, liquid lipids (Capryol™ 90) was used in the preparation of CC-NLC composed of mainly monoesters and a small fraction of diesters of Caprylic/capric triglyceride; therefore, it exhibits a great role and contributes to zeta potential through its polarity, which produced from the non-esterified hydroxyl group of the glycerol and the length of the fatty acids [59,60].

Regarding the surfactant was used as a stabilizer for prepared CC-NLC does not share with an additional charge to zeta potential, due to its non-ionic behavior. Depside of surfactants used has no effect on charge of zeta potential, but possess great impacts on the stability of nanoparticles, due to its steric hindrance nature, which imparts stability to NLC [40].

The high encapsulation efficiency of CC in NLCs could be explained by physicochemical properties of CC, which clearly distinguished in the high lipophilic nature of this molecule (log p ~ 6.2) therefore, enhancing the solubility of CC in various lipids and subsequently highly encapsulated into the lipid matrix [6,8]. Moreover, the use of a combination of highly ordered with less ordered lipids, which form an irregular network of lipid matrix and provided many imperfections leading to the accommodation of a high amount of CC [40,55].

#### 3.2.2. Drug-Excipient Compatibility Studies (By DSC)

Differential scanning calorimeter (DSC) technique was performed widely to investigate the compatibility status of drug and lipids in NLC formulations. Further, to distinguish the interactions between the components [61]. Dependent on the fact that various lipid modifications possess various melting points and enthalpies.

**Figure 1:**
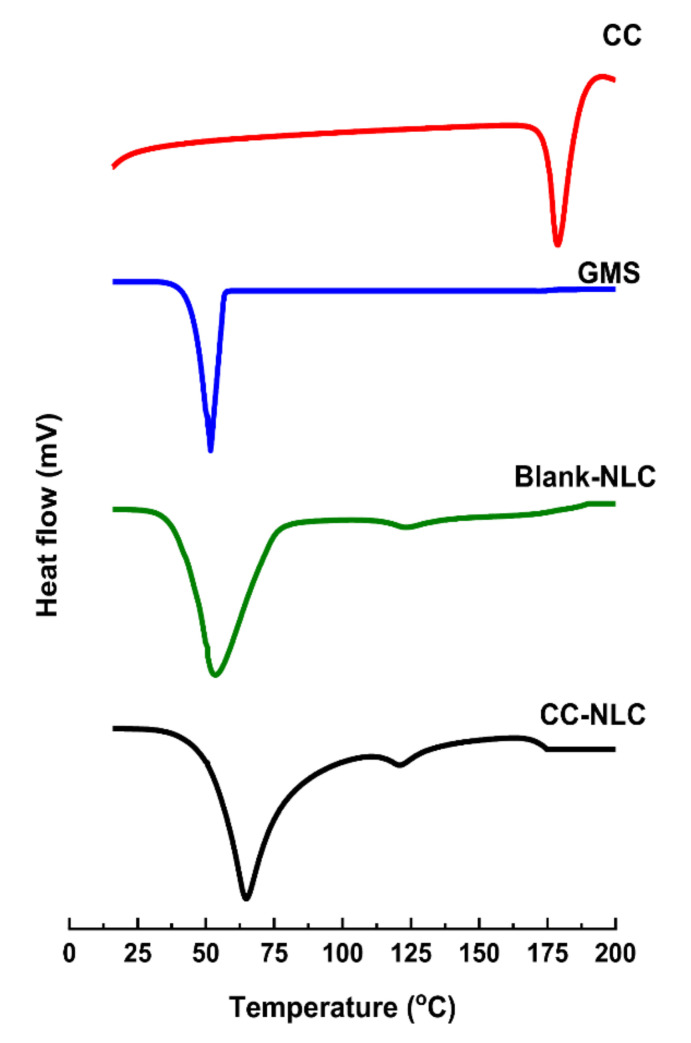
DSC thermograms of CC, GMS, blank-NLC, and CC-NLC.

DSC thermograms of pure drug, GMS, blank-NLC, and CC-NLC formulation are shown in Figure 1.

The DSC thermogram of pure CC showed a sharp endothermic peak at 162.20 °C.

GMS showed sharp endothermic peaks at 51.72 °C. However, blank-NLC and CC-NLC formulation showed a slight shift in temperature of GMS with a sharp endothermic peak at 53.61 °C and 64.69 °C, respectively.

This truth may be attributed to their nanoparticle size, phase dispersion of the lipid, and presence of some excipient as a surfactant [52,62].

On the other hand, the characteristic endotherm peak of CC was not observed in the thermogram of CC-NLC formulation, indicating that conversion of the original crystalline state of the drug to amorphous one. Therefore, CC is completely incorporated and molecularly dispersed in the amorphous state in the lipid matrix [6,54,63].

#### 3.2.3. Fourier Transform Infrared Spectroscopy (FT-IR) Analysis

**Figure 2:**
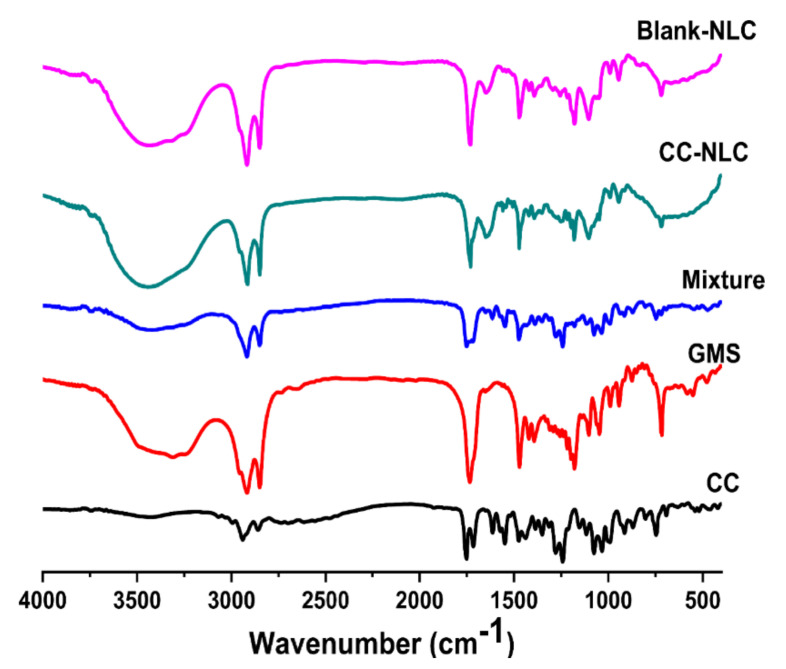
FT-IR spectra of CC, GMS, phys. mixture, CC-NLC, and blank-NLC.

The FT-IR technique was carried out to exclude any physical or chemical interactions that occurred between pure CC and other NLC excipients. The IR spectrum of pure CC, GMS, physical mixture, blank-NLC, and CC-NLC appear in Figure 2.

IR spectrum of CC showed the presence of two characteristic bands for C=O of ester at about 1735 and 1745 cm^−1^ and bands for C–H aliphatic stretching at about 2900–2950 cm^−1^. Besides the presence of a band for C=N aromatic stretching at about 1620 cm^−1^ [2].

IR spectrum of GMS exhibited the presence of a band for C=O of ester at about 1735 cm^−1^ and bands for C–H aliphatic stretching at about 2800–2900 cm^−1^ [64].

The IR spectrum of the physical mixture of CC and GMS showed all bands for each at the same wavenumbers mentioned above. It was found that the band at 1735 was more intense in the mixture, due to the overlapping of certain functional groups for the specific bandwidth [40].

IR spectrum of blank-NLC and CC-NLC revealed the same bands of functional groups, which are carbonyl ester, carboxylic group, and aliphatic C–H in the formula at almost the same wavenumbers.

Regarding the IR spectrum of CC-NLC indicates that no interaction had occurred between the functional groups of the drug (CC) and other components in the formula. Because in the IR spectrum of CC-NLC, all functional groups of CC had bands at the same wavenumbers they had in the IR spectrum of pure CC. If there any interaction, the bands of functional groups would be shifted or disappeared. Furthermore, the similarity in the IR spectrum of both blank-NLC and CC-NLC more confirmation, for there was no physical or chemical interaction between CC and NLC components [41].

#### 3.2.4. Morphology of CC-NLC

**Figure 3:**
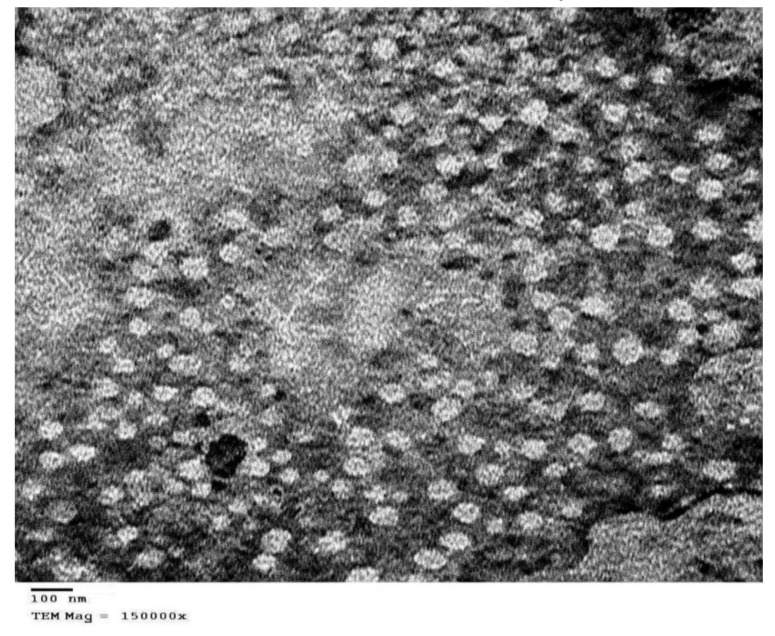
Transmission electron microscope images of CC-NLC.

The morphology and presence of colloidal nanoparticles of CC-NLC were determined by transmission electron microscope and was showed in Figure 3.

TEM images illustrated that CC-NLC was of uniform distribution, separate, and almost spherical shapes. This result might be based on particles developed using lipid with organic polydispersion are almost spherical as in the present study [65].

Whereas, particles developed from highly pure lipids are often cuboid in shape [66]. Also, this fact was agreed with Teeranachaideekul et al. 2007 which stated that The morphology of lipid-based nanocarriers was recorded to be based on the degree of purity of the lipids used in the formulation [60].

The TEM micrograph showed that CC-NLC was nanometer-sized particles around 100 nm, which in proximity with the observations yielded by a Malvern^®^ Zetasizer Nano ZS90 (Malvern^®^ Instruments Limited, Worcestershire, UK), and there were no crystals of the free drug observed.

### 3.3. Storage Stability Study of CC-NLC

Storage stability study was carried out for the developed formula CC-NLC at room temperature almost 25 ± 2 °C and refrigerator which near to 4 ± 2 °C. The particle size, polydispersity index, and zeta potential, as well as encapsulation efficiency, were measured and summarized in Table 2.

Initially, visual inspection has exhibited no agglomeration or separation for CC-NLC during the study period. The obtained results from storage at room temperature clearly showed that increase in particle size and polydispersity index by the end of three months of the study reached 124.60 ± 4.15 nm and 0.43 ± 0.02, respectively, but zeta potential, and EE were decreased to −23.52 ± 0.51 mV and 93.92 ± 4.89%, respectively.

However, only minor changes were observed in mean particle size, polydispersity index, zeta potential, and encapsulation efficiency when the CC-NLC formula stored at refrigerator, where PS and PDI were appeared slight increase to 122.80 ± 5.25 nm and 0.31 ± 0.03, respectively, but zeta potential and entrapment efficiency were lowered to −25.73 ± 0.58 mV and 94.91 ± 3.71%, respectively.

These insignificant changes in the mentioned parameters can be neglected, due to a very small values and may be attributed to some polymorphic changes of the lipid from α less stable polymorph to β stable polymorph a combined by an increase in z-average and polydispersity index and lowering in zeta potential and entrapment efficiency of CC-NLCs [67].

This suggested that CC-NLCs stored either refrigerated or at room temperature are physically stable and do not aggregate or coalesce during storage at both previous conditions.

### 3.4. In Vitro Release Study

In vitro release study was achieved for the prepared formulation in addition to pure CC suspension. The release condition monitored in PBS (pH 6.8) and at the same conditions with adding tween 20 (0.7% *w*/*w*) to achieve “sink” conditions during a dissolution [68].

**Figure 4:**
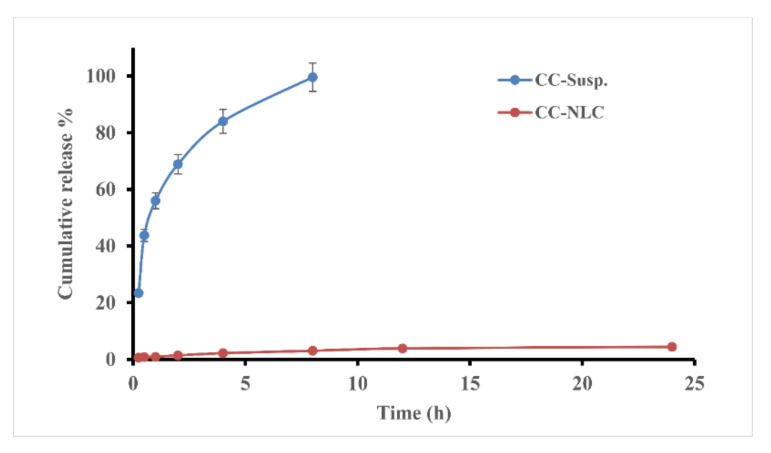
Cumulative release % profile of CC from CC-NLC formulation and CC Suspension in PBS (pH 6.8) with adding Tween 20 (0.7% *w*/*w*).

The in vitro release of CC from developed CC-NLC and CC suspension was graphically represented in Figure 4. It was found that CC-NLC formulation exhibit a lack of drug release. The cumulative drug release of prepared CC-NLC formulation was released less than 5% after 24 h in PBS (pH 6.8) with adding Tween 20 (0.7% *w*/*w*).

Whereas, CC suspension showed almost complete drug release (100%) within 8 h. Because CC has poor solubility (equivalent to 1 μg/mL in PBS pH 6.8) [8] and high lipophilicity (log p ~ 6.2), resulting in very difficult release and high lipid affinity. Therefore, the drug becomes more entrapped and retained inside the core of the lipid matrix, preventing it from release.

Furthermore, the highly efficient solubility and compatibility of CC with the lipid components, as discussed before, in previous a work [32], under screening studies.

These observations are in agreement with the study reported by Reference [8]. Previous studies ascertained that NLCs must be absorbed into the blood or lymphatic system after duodenal administration to rates [29]. Consequently, lack of in vitro release of CC from NLCs, suggesting that NLCs could be absorbed via the enterocytes after oral administration, the most sought-after therapeutic effect.

### 3.5. In Vivo Studies

#### 3.5.1. Absorption of CC-NLC in the Gastrointestinal (GI) Tract

The absorption study of CC-NLC from the stomach and intestine was achieved to get more confirmation about improvement and enhancing the absorption of CC from the gastrointestinal (GI) tract and to determine the main site of absorption of new lipid forms, which can be across any site of (GI) tract. The absorption of CC-NLC from the stomach and intestine was performed compared with that of free-CC suspension.

**Figure 5:**
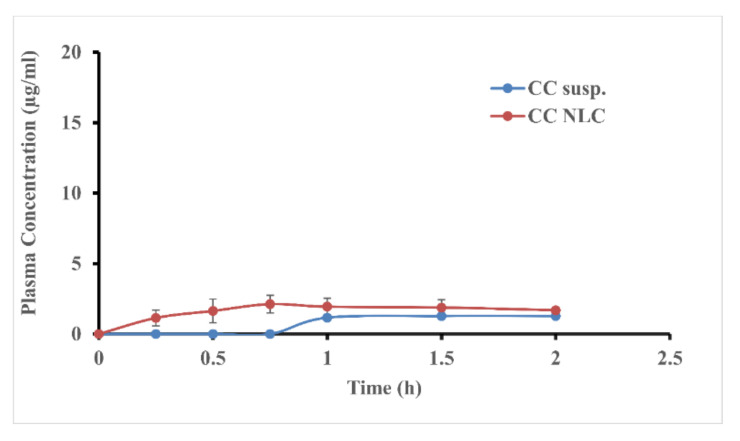
The average plasma concentration of candesartan in rats after intragastric administration of CC-NLC and CC suspension.

**Figure 6:**
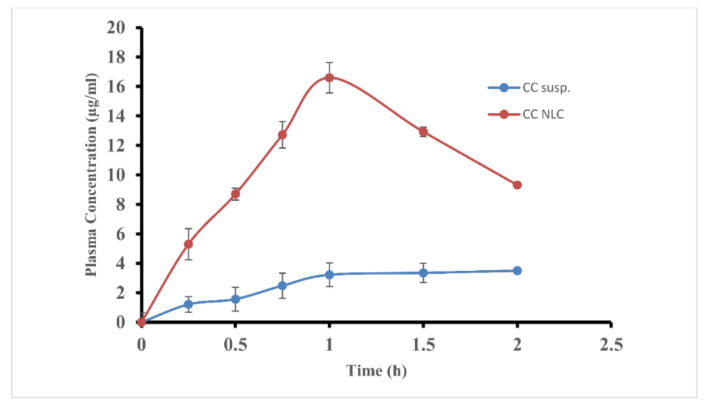
The average plasma concentration of candesartan in rats after intraduodenal administration of CC-NLC and CC suspension.

The data obtained were presented in Figure 5 and Figure 6, which showed that the plasma concentration of candesartan after intragastric or intraduodenal administration was higher than that free CC suspension. AUC(0–t) and C_max_ values of CC-NLC from stomach and intestine were 3.31 ± 0.72, 21.69 ± 1.52 μg·h/mL and 2.12± 1.20, 16.6± 2.40 µg/mL, respectively, which was enhanced around 4.5-fold by comparing with that of the free-CC suspension as illustrated in Table 3.

These results indicated that the encapsulation of CC into NLCs highly improves its absorption from the gastrointestinal (GI) tract. As well as, despite the absorption of CC-NLCs in the stomach was improved, the main site of absorption still was the intestine. Wherein, the absorption of CC-NLC9 in the stomach around 15.26% of that in the intestine.

#### 3.5.2. Cellular Uptake of Fluorescent Blank-NLC

The uptake of Nile red loaded nanostructured lipid carriers (NR-blank-NLC9) by Caco-2 cell monolayer was performed to clarify and confirm its absorption and penetration into the enterocytes.

A hydrophobic fluorescent dye as Nile Red could be loaded into blank-NLC to imagine the internalization of NLCs into enterocytes. Incubation of NR-blank-NLC with Caco-2 monolayer cells are permitted for 3 h to determine the effectiveness of cellular uptake. After 1 h of incubation of fluorescence NR-blank-NLC with colon cancer cells, the fluorescence signal was detected.

**Figure 7:**
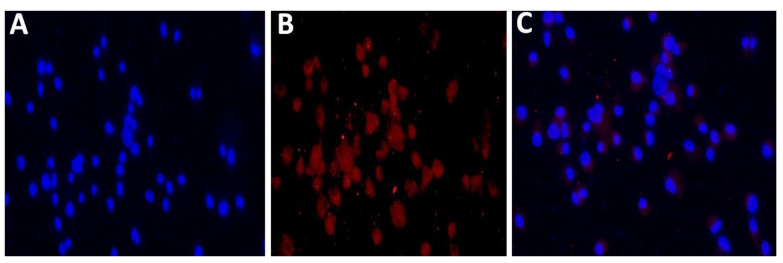
Fluorescence microscopy images of immunofluorescence staining of Caco-2 cell monolayers. Caco-2 cells were incubated with fluorescent NR-NLC for 3 h. The cell nuclei were stained by DAPI (**A**), and the nanoparticles were labeled by Nile Red (**B**). The merge images of the nucleus (blue) and NLCs (red) (**C**). With magnification 40×.

Figure 7 showed the fluorescence microscopic images of (A) nuclei were stained with 4,6-diamino-2-phenylindole DAPI, (B) nanostructured lipid carriers were labeled by Nile Red (NR-blank-NLC) and (C) The merge images of the nucleus (blue) and NR-blank-NLC (red).

The observed results elucidated that high accumulation of fluorescent nanoparticles closed to the nuclei inside the cytoplasm of Caco-2 cancer cells after 3 h of NP incubation [35]. It is believed that fluorescent particles of NR-blank-NLC were taken up by cells via endocytosis, resulting in high cellular accumulation [27,34,69].

The fluorescence signal began to be detectable after 1 h incubation in colon cancer cells, and reaching a maximum at 3 h, when the red signal was predominantly cytoplasmic. This is a very interesting result, since it demonstrates the ability of developed nanoparticles to rapidly penetrate into the cell and transport the loaded drug to the cytoplasm region. These results corroborate a good affinity and biocompatibility between the nanoparticle formulation and cancer cells. These findings, together with the measured pharmacokinetic behavior, revealed the potentiality of NLCs to penetrate the cell and transport the incorporated drug to the cytoplasmic zone.

#### 3.5.3. Pharmacokinetic Behavior of CC-NLC

CC is a prodrug. After oral administration and during absorption from the gastrointestinal (GI) tract it was subjected to rapid ester hydrolysis to produce a therapeutically active candesartan [3,70].

The sufficient therapeutic effect of candesartan should not less than 0.05 μg/mL; therefore, it was essential that the plasma concentration of candesartan should be equal or above 0.05 μg/mL [71].

**Figure 8:**
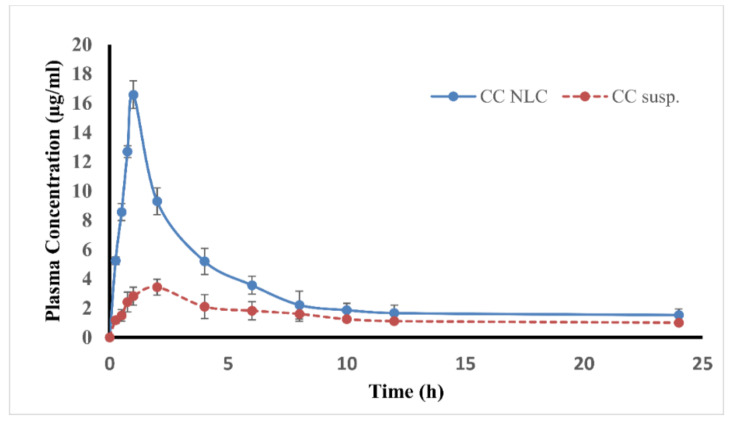
Average plasma concentration vs. time profile following single oral administration of CC-NLC and CC suspension.

The plasma concentration-time profiles after a single oral administration of CC-NLC and free CC suspension were graphically represented in Figure 8, and the basic pharmacokinetic parameters were listed in Table 4.

Based on the obtained results, it was found that the plasma concentration of candesartan in rats treated with CC-NLC was abundantly greater than that of CC suspension. Besides, the average peak plasma concentration (C_max_) of CC-NLC was about 16.58 ± 2.40 μg/mL by noticeable enhancing above 4.5-fold compared to that of the free-CC suspension.

Regarding the area under the curve (AUC_0–t_) of candesartan, which considerable parameter in rats treated with CC-NLC was significantly improved above 2-fold than that of free-CC suspension, this an indicator for increasing and improving the oral bioavailability of candesartan carried by NLCs.

The remarkably enhanced oral absorption of CC-NLC was in accordance with that following the direct administration to the intestine, which indicated that the intestinal absorption was the chief in the improved oral absorption of CC-NLC.

Furthermore, it was observed that the time to reach the maximum concentration C_max_ (t_max_) of candesartan from CC-NLC and the free-CC suspension was 1.00 ± 0.12 h and 2.00 ± 0.14 h, respectively, which showed that the encapsulation of CC in NLCs get it absorbed more quickly.

The therapeutic effect of CC was achieved at 4–6 h from free-CC tablets following oral use [3]. The rapid absorption of CC-NLC than that free CC suspension could be useful in treating high pressure or heart failure, especially in clinical therapeutic emergencies.

Finally, the elimination half-life (t_1/2_) for developed CC-NLC and free CC suspension was 19.65 ± 2.18 h and 9.51 ± 1.15 h, respectively, and mean residence time (MIR) was found in CC-NLC higher than that in free CC suspension 15.99 ± 4.20 h and 10.79 ± 3.10 h, respectively.

The in vivo pharmacokinetic behavior, which was based on the components and properties of CC-NLC, showed that the NLC can improve and enhance the oral bioavailability of CC by one or more of the following mechanisms [6,72].

First, the high tendency of mucoadhesion of CC-NLCs to the intestinal wall. Second, surface-active properties of surfactants used in formulation enhance the permeability and solubility of the drug through the gastrointestinal tract membrane. Third, the nanoparticle size of CC-NLCs contributes to the enterocyte’s surface to increase. Consequently, improve and enhance drug permeability across the intestinal membrane [27,28]. Meanwhile, the nanometric size of CC-NLCs adhere to the gastrointestinal tract easily for a long period and to intervillar spaces lead to the mean residence time to increase associated with the increased bioavailability of CC.

Fourth, long carbon chain fatty acids of solid lipids used in CC-NLCs formulation had an important role and more favor the lymphatic transport through chylomicron formation [16,17,29,30] leads to avoidance of the first-pass metabolism, thus, increased bioavailability of CC [31].

From the introduced discussion, it can be concluded that the absorption of designed CC-NLC was more rapid than that of free CC suspension.

Further, the oral bioavailability of CC was enhanced to above 2-fold. Thus, NLCs provide a potential approach to enhance and improve the oral bioavailability of CC.

## 4. Conclusions

In current work, CC-NLC were successfully prepared using a hot homogenization-ultrasonication technique. The obtained CC-NLCs showed small and homogeneous particle size with high entrapment efficiency.

The physicochemical characterization indicated the amorphous state of CC followed incorporation into the NLCs delivery system. The pharmacokinetic behavior and storage stability were performed for the developed formulation.

The pharmacokinetic results appeared that the oral bioavailability of CC was remarkably augmented above 200 percent after incorporation into nanostructured lipid carriers.

The absorption study of CC-NLCs in the gastrointestinal (GI) tract revealed that CC-NLCs absorption mainly occurred in the intestine.

Moreover, CC-NLCs must be internalized into enterocytes and transported into systemic circulation through intestinal lymphatic transportation, as confirmed in the uptake of CC-NLCs by Caco-2 cancer cells.

From this study, it can be concluded that nanostructured lipid carriers offered high beneficial effect for enhancing and improving the oral bioavailability of poorly aqueous soluble drugs, such as CC.

## Figures and Tables

**Table 1:** Composition and physicochemical characterization of Candesartan Cilexetil (CC) successfully encapsulated into a nanostructured lipid carrier (NLP).

Formula Composition	
Lipid Phase	
Solid lipids 70% of lipids	GMS
Liquid lipids 30% of lipids	Capryol™ 90
Drug = 5% of total lipids (*w*/*w*)	CC
**Aqueous Phase**	
Surfactants 2.5% (*w*/*w*) by ratio (1:1)	Lutrol^®^ F127: Cremophor^®^ RH
Water	Water
**Physicochemical Characterization**	
Particle size (PZ)	121.6 ± 6.2 nm
Poly dispersity index (PDI)	0.26 ± 0.03
Zeta potential (ZP)	−26.5 ± 2.9 mV
Encapsulation efficiency (EE)	96.23 ± 3.14%

**Table 2:** Storage stability study of CC-NLC; ^NS^: Not significantly difference at p > 0.05.

Storage Condition	Size ± SD (nm)	PDI ± SD	Zeta ± SD (mV)	EE ± SD%	Visual Observation
**Fresh**	121.6 ± 6.20	0.26 ± 0.03	−26.5 ± 2.90	96.23 ± 3.14	Clear emulsion
**Refrigerator**					
**1 month**	121.8 ± 7.23	0.28 ± 0.02	−26.1 ± 2.05	95.86 ± 3.91	Clear emulsion
**2 months**	122.1 ± 4.44	0.30 ± 0.05	−25.91 ± 3.02	95.38 ± 4.71	Clear emulsion
**3 months**	122.8 ± 5.25 ^NS^	0.31 ± 0.03 ^NS^	−25.73 ± 0.58 ^NS^	94.91 ± 3.71 ^NS^	Clear emulsion
**Room Temperature (25 °C, RH 65%)**					
**1 month**	122.5 ± 6.23	0.33 ± 0.03	−25.2 ± 4.45	94.84 ± 5.91	Clear emulsion
**2 months**	123.7 ± 8.13	0.39 ± 0.02	−24.3 ± 5.23	94.10 ± 6.75	Clear emulsion
**3 months**	124.6 ± 4.15 ^NS^	0.43 ± 0.02 ^NS^	−23.52 ± 0.51 ^NS^	93.92 ± 4.59 ^NS^	Clear emulsion

**Table 3:** Pharmacokinetic parameters of CC in rats following the absorption of CC-NLC from the stomach and intestine at a dose of 10 mg/kg.

Parameters	Oral Administration of CC-NLC (10 mg/kg) Stomach	Oral Administration of CC-NLC (10 mg/kg) Intestine
**AUC _0–end_ (µg h/mL)**	15.56 ± 5.65	35.87 ± 4.24 *
**AUC _0–t_ (µg h/mL)**	3.31 ± 0.72	21.69 ± 1.52 ***
**C_max_ (µg/mL)**	2.12 ± 1.20	16.6 ± 2.40 ***
**t_max_ (h)**	0.75 ± 0.12	1.00 ± 0.14
**t½ (h)**	5.02 ± 1.02	1.05 ± 1.03 *
**MRT (h)**	1.80 ± 0.26	1.46 ± 0.68
**F_rela_ (%)**	-	655.28

* Significantly difference at *p* < 0.05; *** Extremely significant difference at *p* < 0.0001.

**Table 4:** Pharmacokinetic parameters of CC in rats following oral administration of CC suspension and CC-NLC at a dose of 10 mg/kg.

Parameters	Oral Administration of CC Suspension (10 mg/kg)	Oral Administration of CC-NLC9 (10 mg/kg)
**AUC _0–end_ (µg h/mL)**	64.35 ± 3.20	98.53 ± 4.20 ***
**AUC _0–t_ (µg h/mL)**	35.70 ± 2.10	77.52 ± 3.40 ***
**C_max_ (µg/mL)**	3.44 ± 1.20	16.58 ± 2.40 **
**t_max_ (h)**	2.00 ± 0.14	1.00 ± 0.12 **
**t½ (h)**	9.51 ± 1.15	19.65 ± 2.18
**MRT (h)**	10.79 ± 3.10	15.99 ± 4.20
**F_rela_**	-	217.14

** High Significant difference at *p* < 0.001; *** Extremely significant difference at *p* < 0.0001.
